# Genome-wide association analysis of nine reproduction and morphological traits in three goat breeds from Southern China

**DOI:** 10.5713/ab.21.0577

**Published:** 2022-06-24

**Authors:** Xiaoyan Sun, Jing Jiang, Gaofu Wang, Peng Zhou, Jie Li, Cancan Chen, Liangjia Liu, Nianfu Li, Yuanyou Xia, Hangxing Ren

**Affiliations:** 1 Chongqing Academy of Animal Sciences, Chongqing, 402460, China; 2 Chongqing Engineering Research Center for Goats, Chongqing, 402460, China; 3 Youyang County Livestock Industry Development Center, Chongqing, 409800, China

**Keywords:** Genome-wide Association Study, Goat, Morphological Traits, Reproduction Traits

## Abstract

**Objective:**

This study aimed to investigate the significant single nucleotide polymorphisms (SNPs) and genes associated with nine reproduction and morphological traits in three breed populations of Chinese goats.

**Methods:**

The genome-wide association of nine reproduction and morphological traits (litter size, nipple number, wattle, skin color, coat color, black dorsal line, beard, beard length, and hind leg hair) were analyzed in three Chinese native goat breeds (n = 336) using an Illumina Goat SNP50 Beadchip.

**Results:**

A total of 17 genome-wide or chromosome-wide significant SNPs associated with one reproduction trait (litter size) and six morphological traits (wattle, coat color, black dorsal line, beard, beard length, and hind leg hair) were identified in three Chinese native goat breeds, and the candidate genes were annotated. The significant SNPs and corresponding putative candidate genes for each trait are as follows: two SNPs located on chromosomes 6 (*CSN3*) and 24 (*TCF4*) for litter size trait; two SNPs located on chromosome 9 (*KATNA1*) and 1 (*UBASH3A*) for wattle trait; three SNPs located on chromosome 26 (*SORCS3*), 24 (*DYM*), and 20 (*PDE4D*) for coat color trait; two SNPs located on chromosome 18 (*TCF25*) and 15 (*CLMP*) for black dorsal line trait; four SNPs located on chromosome 8, 2 (*PAX3*), 5 (*PIK3C2G*), and 28 (*PLA2G12B* and *OIT3*) for beard trait; one SNP located on chromosome 18 (*KCNG4*) for beard length trait; three SNPs located on chromosome 17 (*GLRB* and *GRIA2*), 28 (*PGBD5*), and 4 for hind leg hair trait. In contrast, there were no SNPs identified for nipple number and skin color.

**Conclusion:**

The significant SNPs or genes identified in this study provided novel insights into the genetic mechanism underlying important reproduction and morphological traits of three local goat breeds in Southern China as well as further potential applications for breeding goats.

## INTRODUCTION

The economic efficiency of the goat industry is directly affected by reproductive traits, among which the litter size and nipple number are essential parameters for evaluating the reproductive performance of female goats. Improving the litter size has particularly aroused the interest of husbandry enterprises and breeders, as even a slight increase in the litter sizes in goats can considerably profit the breeders. Meanwhile, including morphological traits in the selection criteria is mainly based on their relationships with the other economic traits of goats and sheep [1].

Genome-wide association studies (GWAS) have been widely used as an effective strategy for identifying single nucleotide polymorphisms (SNPs) associated with the traits of economic interest in livestock. The Banjiao goats, Hechuan white goats, and Youzhou dark goats are important indigenous goat breeds and characteristic genetic resources in Southern China, as well as an essential part of the goat industry in Chongqing province [2]. In the past few years, various efforts have been made for identifying the genetic variations in the reproduction traits, e.g. litter size [3,4] and nipple number [5–7], morphological traits, e.g. coat color [8], skin color [9,10], wool [11,12], and wattle [13] in different sheep and goat breeds by GWAS, which has led to the identification of different candidate genes or SNPs. However, the lack of consistency among these findings has suggested the complexity of the genetic basis of these traits. Despite the importance of reproduction and morphological traits, the underlying genetic mechanisms have not been fully elucidated and have consequently hindered the application of marker-assisted selection in three Chinese native goat breeds. Therefore, there is an urgent need for identifying the candidate genes likely responsible for these traits to improve the efficiency of goat production.

This study has aimed in investigating the putative candi date genes and SNPs underlying the genetic mechanism of two reproduction traits (litter size, nipple number) and seven morphological traits (wattle, skin color, coat color, black dorsal line, beard, beard length, and hind leg hair) in three Chinese goat breed populations. Our study might provide new insights into the molecular underpinnings of these traits in Chinese native goat breeds, thereby providing a basis for further exploring its role in goat breeding.

## MATERIALS AND METHODS

### Sampling and genotyping quality control

The blood samples were obtained from 336 unrelated adult goats of three breeds (Hechuan white goat, Banjiao goat, and Youzhou dark goat) in Chongqing province, southwest China. The litter size and nipple number traits were analyzed only on the data of female goats. The experimental procedure involved animals was approved by the ethics committee of the Chongqing Academy of animal sciences (Approval Number: xky-20180716). The individuals were grouped into case and control for nine traits, as denoted in Table 1. The genomic DNA was isolated and purified from whole blood using QIAamp DNA Blood Mini Kit (Qiagen, Hilden, Germany). The qualified DNA was genotyped using the Illumina Goat SNP50 BeadChip panel [14] (Illumina Inc., San Diego, CA, USA), following the manufacturer’s instructions.

After genotyping, the quality control of the genotypes, phenotypic data, and samples was conducted using PLINK 1.07 [15]. The SNPs were analyzed, and the genotypes and phenotypic values were estimated. The SNP markers which fulfilled the following quality control criteria such as i) sample call rate >90%, ii) SNP call rate >90%, iii) a minor allele frequency (MAF) of >5% per SNP, iv) Hardy-Weinberg equilibrium (HWE) >0.000001, were primarily considered. A total of 41,784 SNP markers from 320 samples have remained for further association analyses following quality control.

### Genome-wide association studies

Based on a case-control design for nine traits, the GWAS were analyzed using a generalized linear model implemented in the package GenAbel from R [16]. The corrected significant p-values were used for analyzing the subsequent association. The population structure was analyzed using ADMIXTURE 1.3 [17]. The quantile-quantile plots (Q-Q plots) and Manhattan plots from GWAS analyses were produced using the ggplot2 in the R package. The suggestive genome-wide association significance threshold was p<1.20×10^−6^ (0.05/41784), and the chromosome-wide significance level threshold was p<3.58×10^−5^ (0.05/41784/30), as referred to Wang et al [11]. The most significant SNPs per trait were selected as the lead SNPs, and the candidate genes were further searched. The identified SNPs were annotated to the *Capra hircus* reference genome GCF-001704415.1 (ARS1) within approximately 100 KB upstream and downstream regions.

## RESULTS AND DISCUSSION

**Figure 1:**
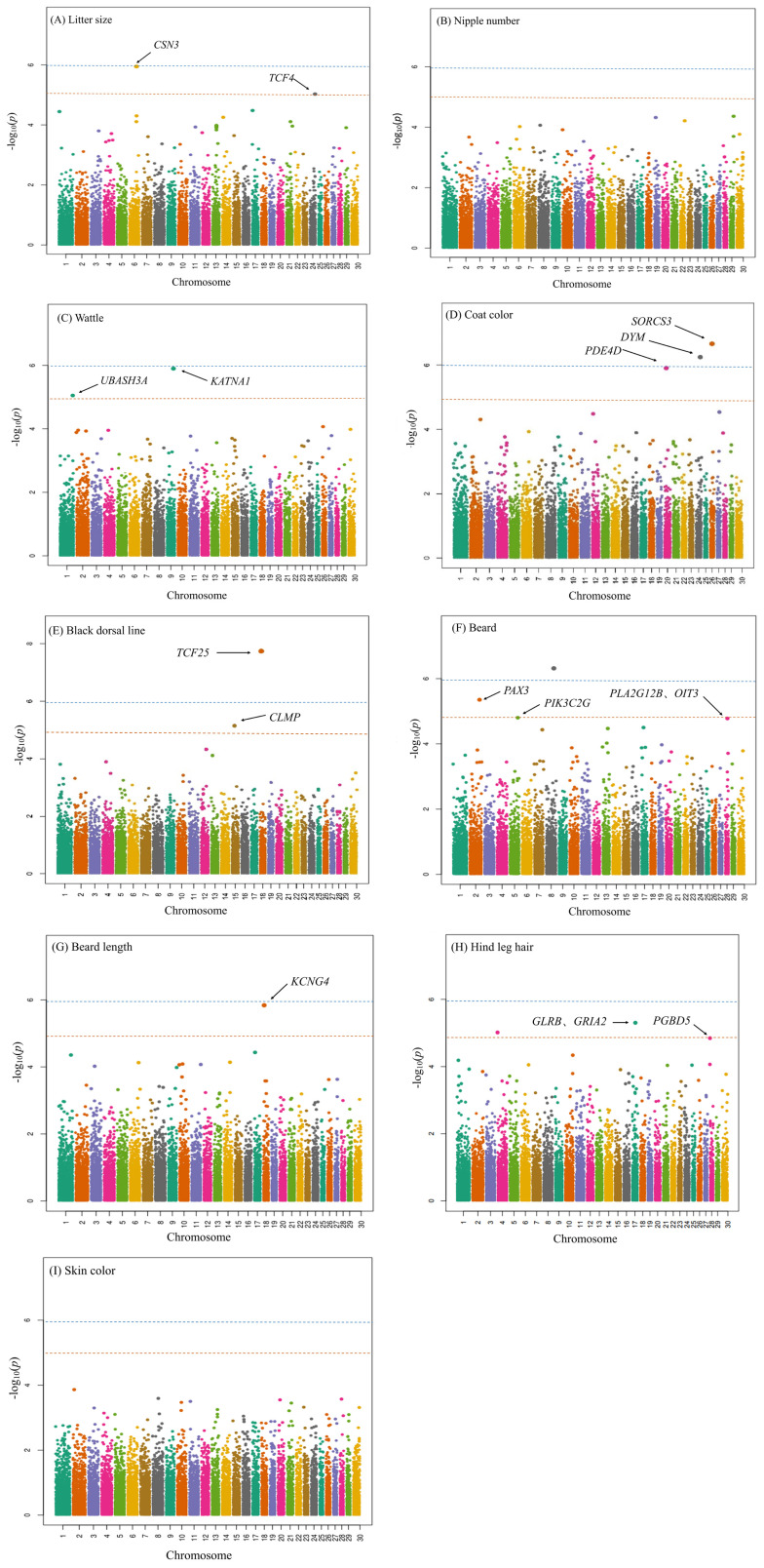
Manhattan plots showing the genome-wide and chromosome-wide distribution of SNPs for seven reproduction and morphological traits in three Chinese native goat breeds (A, litter size; B, nipple number; C, wattle; D, coat color; E, black dorsal line; F, beard; G, beard length; H, hind leg hair; I, Skin color). The blue and orange horizontal lines indicate the thresholds for genome-wide and chromosome-wide significance levels, respectively. Manhattan plots showing the genome-wide and chromosome-wide distribution of SNPs for seven reproduction and morphological traits in three Chinese native goat breeds (A, litter size; B, nipple number; C, wattle; D, coat color; E, black dorsal line; F, beard; G, beard length; H, hind leg hair; I, Skin color). The blue and orange horizontal lines indicate the thresholds for genome-wide and chromosome-wide significance levels, respectively.

The Q-Q plots reasonably confirmed the distribution of the p-value (Supplementary Figure S1) and suggested no genomic inflation or systematic bias in our study. All the SNPs that were significantly associated with each trait were annotated at the genome-wide and chromosome-wide levels. Then, the candidate genes that harbored the most significant SNPs for each trait were discussed. The GWAS results were visualized using the Manhattan plots (Figure 1) and summarized in Table 2.

### Reproduction traits

#### Litter size

Two significant SNPs were found associated with the litter size: an SNP located in the genomic region on chromosome 6 (casein kappa [CSN3]) at the genome-wide level, and another SNP located in the genomic region on chromosome 24 (transcription factor 4 [TCF4]) at the chromosome-wide level (Figure 1A). The CSN3 gene, which encodes a major milk protein known as kappa-casein, plays a vital role in protein synthesis and stability of casein micelle. Another study suggests this gene is an essential putative candidate gene for milk-producing traits in different dairy cow and goat breeds [18]. Lan et al [19] demonstrated that *CSN3-Taq*I locus was associated with litter size in Saanen dairy goats, which is consistent with our result. In addition, several candidate genes involved in this trait have been identified in other goat populations. Previously, Lan et al [20] reported two SNPs to be significantly associated with the litter size in Yunnan black goats, located downstream of the solute carrier family 4 member 10 (*SLC4A10*) gene and upstream of the T-box brain transcription factor 1 (*TBR1*) gene, respectively. Islam et al [21] identified several candidate genes like meiosis arrest female 1 (*MARF1*), synaptonemal complex protein 2 (*SYCP2*), transmembrane protein 200C (*TMEM200C*), steroidogenic factor-1 (*SF1*), adenylate cyclase 1 (*ADCY1*), and bone morphogenetic protein 5 (*BMP5*) involved in goat fecundity in Chinese goat populations. In another study, the KiSS-1 metastasis suppressor (*KISS1*) gene was related to the litter size in the Chinese Arbas Cashmere goat [3], coincident with the findings from the Black Bengal goat and Jining grey goat. Genome-wide screening in the Yunshang black goat identified some loci like gap junction protein beta 2 (*GJB2*), gap junction protein beta 6 (*GJB6*), stabilin 2 (*STAB2*), and heparan sulfate-glucosamine 3-sulfotransferase 2 (*HS3ST2*) to significantly affect the litter size [22]. Most recently, a genome-wide selective sweep analysis has revealed that the insertion/deletion variants within the insulin-like growth factor 2 mRNA-binding protein 2 (*IGF2BP2*) gene were correlated with the litter size in Shaanbei white Cashmere goat [23].

The above research results demonstrated various efforts for identifying the causal genes responsible for litter size in goats, but the results vary among breeds. These inconsistent findings might suggest that the complex genetic mechanisms underpin litter size variation. Our results provided a helpful reference for explaining the genetic mechanism of litter size in goats.

#### Nipple number

Nipples (or teats) are epidermal appendages on the udders or breasts of mammals. Nipple number is a valuable trait crucial to female goat reproduction performance and offspring growth [6]. Sheep and goats normally have only two functional nipples, whereas a small number of female goats under investigation possess one or two supernumerary nipples. Studies suggested that supernumerary nipples might be susceptible to bacterial ingress through the teat canal and consequently impede their production efficiency considerably [24]. Therefore, the supernumerary nipples in goats and sheep are undesirable.

In this study, no SNPs were identified for nipple number trait (Figure 1B). The BBX high mobility group box domain containing (*BBX*) and CD47 molecule (*CD47*) gene has been reported to be significantly linked to the supernumerary nipple phenotype in Wadi sheep [5]. Additionally, another recent genome-wide comparative analysis has shown the γ-aminobutyric acid type A receptor subunit γ3 (*GABRG3*), LDL receptor related protein 1B (*LRP1B*), and mono-ADP ribosylhydrolase 2 (*MACROD2*) genes to be associated with the nipple numbers in Luzhong mutton sheep [25]. Furthermore, there was noteworthy relevance of nipple number and reproduction traits, e.g., litter size in mammals such as pigs and goats [6], suggesting that further research could combine both the nipple number and litter size trait for seeking candidate genes. Nevertheless, a GWAS on Saanen dairy goat illustrated 17 regions on ten chromosomes to be significantly associated with the supernumerary nipple trait at the chromosome-wide level. In contrast, there were no SNPs at the genome-wide level. It suggested that supernumerary nipple trait might be inherited in a polygenic fashion, consistent with the recent findings in cattle [7]. Therefore, the failure in identifying the SNPs for the nipple number trait implied that other causal loci and feasible mechanisms might exist in the three Chinese native goat populations.

### Morphological traits

#### Wattle

Congenital appendages (wattles) are commonly found on the throat of goats and are reported to be under the genetic control by the *W* gene with an inheritance. Previous studies reported that female goats with wattle have 7% higher fecundity than those without wattle, and highlighted the importance of this trait in marker-assisted breeding.

At the chromosome-wide level, two significant SNPs were identified for the wattle trait located in the genomic regions on chromosomes 9 (katanin catalytic subunit A1 [*KATNA1*]) and 1 (ubiquitin associated and SH3 domain containing A [*UBASH3A*]) (Figure 1C). Katanin is a heterodimer microtubule-severing enzyme comprising a catalytic p60 subunit A1 and an 80 kDa regulatory p80 subunit B1, in which *KATNA1* encodes the p60 subunit A. It is also involved in regulating neuronal progenitor proliferation *in vivo* during embryonic development and adult neurogenesis. On the other hand, the *USBSH3A* gene encodes one of the two family members belonging to the T-cell ubiquitin ligand which can negatively regulate the T-cell signaling. Previous studies have indicated that genetic variations in the *UBASH3A* gene were associated with several autoimmune diseases such as diabetes, rheumatoid arthritis, and systemic lupus erythematosus.

Earlier, a GWAS performed in nine different Swiss goat breeds identified two significant SNPs on chromosome 9 which contained the formin 1 (*FMN1*) and gremlin 1 (*GREM1*) genes [13], responsible for limb development and outgrowth. These findings supported earlier assumptions that wattles are rudimentary developed extremities. Also, this is the first study demonstrating the associations between the *KATNA1* and *UBASH3A* genes with wattle in goats based on our current knowledge.

#### Coat color

The genetic determinants of goat coat color are not only important for improving the artificial selection efficiency of the desired coat color but also crucial for understanding the different evolutionary dynamics in shaping the phenotypic variation in farm and wild animals. Two significant SNPs were identified for coat color trait (white vs brown) at the genome-wide level and were located in the genomic regions on chromosomes 26 (sortilin-related VPS10 domain-containing receptor 3 [*SORCS3*]) and 24 (Golgi-associated protein Dymeclin [*DYM*]). On the other hand, at the chromosome-wide level, one significant SNP was identified for the coat color trait located in the genomic regions on chromosome 20 (phosphodiesterase 4D [*PDE4D*]) (Figure 1D).

The *SORCS3* gene encodes a type-I receptor transmembrane protein having a vital role as a sorting agency within the cells and transports various intracellular proteins. The *DYM* gene encodes a Golgi-associated protein (Dymeclin), whose deficiency typically leads to Dyggve-Melchior-Clausen syndrome (DMC). The melanosomes are discontinuous membranous organelles formed by the Golgi apparatus-endoplasmic reticulum-lysosome, and they are the only organelles synthesizing and depositing melanin in the eukaryotic cells. In addition, the studies in mice and broilers revealed the *SORCS3* gene to affect feed efficiency, energy balance, and orexigenic peptide production and suggested a relationship between pigmentation and energy metabolism [26,27]. Thus, it is most probable that *SORCS3* and *DYM* have essential roles in the variation of coat color in goats in the present study. Moreover, the *PDE4D* gene, encodes the phosphodiesterase 4D having a 3′,5′-cyclic-AMP phosphodiesterase activity, with an essential role in signaling pathways of melanin synthesis. Although well-known genes like agouti signaling protein (*ASIP*), tyrosinase related protein 1 (*TYRP1*), melanocyte inducing transcription factor (*MITF*), melanocortin 1 receptor (*MC1R*), and KIT proto-oncogene (*KIT*) have been associated with coat color in various goat populations [28], our results provided a new variation source of coat color in goats.

#### Black dorsal line

The color pattern is important for both wild and domesticated animals. The black dorsal line is a unique coat color pattern in goats and many animals such as Chinese hamsters, Sugar gliders, and slow loris. The genetic basis of this trait mechanism remains unclear.

At the genome-wide level and chromosome-wide level, two significant SNPs were identified for the black dorsal line trait located in the genomic regions on chromosome 18 (transcription factor 25 [*TCF25*]) and 15 (CXADR like membrane protein [*CLMP*]), respectively (Figure 1E). The T cell transcription factors (TCF) are essential modulators for Wnt-induced transcriptional activation, with essential roles in cell fate decisions during embryonic development and stem cell homeostasis in somatic niches of normal tissues. The TCF transcription factors participate in melanoma progression by regulating the versican expression. GWAS by Wang et al [29] demonstrated that TCF25 might be a putative candidate gene for coat color in Tan sheep. In mouse embryos, TCF25 is intensely expressed in the dorsal root ganglia. For this reason, we assume that the regional differential expression of TCF25 might modulate the subsequent melanocyte migration leading to the dark middorsal strip in goats. The previous investigation on the *CLMP* gene has reported this gene to be responsible for encoding the CXADR-like membrane protein localized to the junctional complexes between the endothelial and epithelial cells and might have a role in cell-cell adhesion. The expression of the *CLMP* gene in the human white adipose tissue is implicated in adipocyte maturation and obesity development. Therefore, the association between the black dorsal line and the *CLMP* gene remains yet to be verified.

#### Beard

Beard is a qualitative trait that significantly benefits the survivability, climate adaptability, and productivity of goats in tropical regions. The beard traits have been evaluated in some goat breeds [2]. However, the underlying molecular mechanism needs more clarification.

At the genome-wide level, one significant SNP was identi fied for beard trait on chromosome 8, but no candidate gene was found. While at the chromosome-wide level, three significant SNPs were identified for beard trait in the genomic regions on chromosome 2 (*PAX3*), 5 (*PIK3C2G*), and 28 (*PLA2G12B* and *OIT3*) (Figure 1F). The *PAX3* gene, which encodes a member of the paired box family of transcription factor 3 (PAX3), is critical for developing melanoblasts. The *PIK3C2G* gene encodes phosphatidylinositol-4-phosphate 3-kinase catalytic subunit type 2 gamma, associated with cell migration. According to other reports, the protein encoded by the *PLA2G12B* gene belongs to the phospholipase A2 (PLA2) group of enzymes and might function as a negative regulator of tumor progression. While the *OIT3* gene encoding the oncoprotein-induced transcript 3 is a novel biomarker of alternatively activated macrophages and facilitates hepatocellular carcinoma metastasis.

Previously, a GWAS performed on the Beijing fatty chicken showed that the copy number variations in the homeobox B7 (*HOXB7*) and homeobox B8 (*HOXB8*) genes were involved in forming beard traits [30]. While in goats, evaluating the beard traits has been carried out in some goat breeds in Southwest China recently [2], but no previous studies have so far investigated the goat beard traits by genome selection approach. To our knowledge, this study attempted to study the candidate genes or SNPs associated with goat beard traits using GWAS for the first time, and our results might provide a potential phenotypic selection strategy for goat breeding in Southwest China.

#### Beard length

Some of the goats under investigation were found to have long beards while others have short beards. A study in anestrus dairy goats evaluated some morphometric traits including beard length, for exploring the possible role of these traits in goat reproductive success [31]. Such morphometric advantages shown by the high social rank goats emphasized the need for comprehending the biological basis of relevant animal traits and yet, the genetic mechanism of beard length remains unclear. Therefore, exploring the genetic basis of beard length traits could facilitate the sustainable utilization, conservation, and improvement of the genetic resources in goats.

At the genome-wide level, one significant SNP was identi fied for the beard length trait and was located in the genomic regions on chromosome 18 (*KCNG4*) (Figure 1G). The *KCNG4* gene encodes the potassium voltage-gated channel modifier subfamily G member 4, functioning as a modulatory subunit. A previous study also identified the *KCNG4* gene as a candidate gene for multiple sclerosis in humans. This study could be the first one to report that the *KCNG4* gene is associated with the beard length trait in goats, and the results could also help in revealing the genetic mechanism of beard length traits in goats.

#### Hind leg hair

Genetic improvement of hair/wool-related features is a major goal in the goat and sheep industry, while hind leg hair is associated with the hair/wool quality and quantity in goats. Although the genetic variation of hair/wool traits has been explored in sheep and goats [8], the genetic architecture controlling the wool traits has not been fully elucidated. Therefore, a comprehensive understanding of the molecular genetic mechanisms of wool traits is needed for improving the breeding efficiency of goats.

At the chromosome-wide level, three significant SNPs were identified for the hind leg hair traits. One of the identified SNPs is located on chromosome 17 (between *GLRB* and *GRIA2*); the other SNP is located on chromosome 28 (*PGBD5*); the third SNP is located on chromosome 4 but not annotated to any known gene (Figure 1H). The *GLRB* gene encodes the glycine receptor β subunit with an important role in binding agonists and conducting ion channels. The missense mutations in the glycine receptor β subunit might underlie disease pathology in the human startle disease. The *GRIA2* gene encodes glutamate ionotropic receptor AMPA type subunit 2, which is a predominant excitatory neurotransmitter receptor in the mammalian brain. A recent study showed that the de-novo variants in the *GRIA2* gene could cause neurodevelopmental disorders in humans. The *PGBD5* gene encodes the piggyBac transposable element derived protein 5, which belongs to the group of transposases. According to other reports, the *PGBD5* gene could also promote site-specific oncogenic mutations in human solid tumors. Furthermore, a previous GWAS performed on yearling Merino sheep identified that the fin bud initiation factor homolog (*FIBIN*), hydroxysteroid 17-beta dehydrogenase 11 (*HSD17B11*), and protein inhibitor of activated STAT 1 (*PIAS1*) genes are related to the development and growth of hair/wool, which is inconsiderate with the genes identified in our study. To date, our study could be the first to report that the *GLRB*, *GRIA2*, and *PGBD5* genes are associated with the hind leg hair traits in goats, and the results could also help to reveal the genetic mechanism of hair/wool traits in goats.

#### Skin color

In some sheep breeds, black skin spots (skin pigmentation) can also produce pigmented fibers which significantly decrease the fiber quality. Therefore, elucidating the genetic mechanism of skin color in goats and sheep is of great economic value.

In this study, no SNPs were identified for the skin color traits (Figure 1I). Earlier studies have reported that the *TYRP1*, FBJ murine osteosarcoma viral oncogene homolog (*C-FOS*), Krüppel-like factor 4 (*KLF4*), ubiquitin-fold modifier conjugating enzyme 1 (*UFC1*), and *ASIP* genes are associated with skin pigmentation in sheep and goats [32,33]. However, the genetic mechanism underlying skin color traits remains unclear. For livestock, a majority of complex traits are likely controlled by a larger number of genes that individually confer a small effect. Furthermore, a GWAS performed on the Hereford cattle provided evidence that eyelid pigmentation is a heritable trait influenced by many loci [12]. Moreover, given that most tests for association proceed in a simple SNP-by-trait fashion using stringent significance levels to offset multiple testing, many of the genes contributing to trait variation remain undetected [33]. However, in a study performed on the pigmentation phenotype (piebald) in Merino sheep, insulin-like growth factor binding protein 7 (*IGFBP7*), platelet-derived growth factor alpha (*PDGFRA*), and CD9 molecule (*CD9*) were identified as gene drivers for the piebald phenotype by combing GWAS with gene expression data [10]. This study was implicated in exploring approaches that sought to incorporate GWAS into systems biology, such as considering merging SNP variation with the analyses of differential gene expression for investigating the basis of a skin pigmentation phenotypic trait.

## CONCLUSION

Several potential candidate genes or SNPs associated with a reproduction trait (litter size) and six morphological traits (wattle, coat color, black dorsal line, beard, beard length, and hind leg hair) were identified in three Chinese native goat breed populations. These findings will contribute to a better understanding of the genetic basis of reproduction and morphological traits and provide breeding strategies for goat production.

## Figures and Tables

**Table 1:** The grouping information of nine reproduction and morphological traits in three Chinese native goat breeds

Trait name	Samples	

	Case	Control
Litter size	More than 2 lambs	1 to 2 lambs
Nipple number	More than 2 nipples (supernumerary)	Only 2 nipples
Wattle^1)^	Present	Absent
Skin color	Pigmented	Unpigmented
Coat color	Brown	White
Black dorsal line^2)^	Present	Absent
Beard	Present	Absent
Beard length	Long	Short
Hind leg hair	Long	Short

1) A pair of fleshy pendants on either side of the jaw.

2) A dark stripe at the mid-dorsal region.

**Table 2:** Details on significant SNPs (genome- and chromosome-wide), and the annotated genes for seven reproduction and morphological traits in three Chinese native goat breeds

Chr	SNP marker name	SNP marker position (bp)	Significance	Annotated gene (p-value)	Significance type	Trait
1	snp40293-scaffold514-1068053	140,320,986	8.86E-06	*UBASH3A* (ubiquitin associated and SH3 domain containing A)	CW	Wattle
2	snp7450-scaffold127-3029568	109,852,935	4.45E-06	*PAX3* (paired box 3)	CW	Beard
4	snp46403-scaffold641-657922	657,922	9.67E-06	-	CW	Hind leg hair
5	snp43885-scaffold593-645533	92,866,491	1.58 E-05	*PIK3C2G* (hosphatidylinositol-4-phosphate 3-kinase catalytic subunit type 2 gamma)	CW	Beard
6	snp59472-scaffold980-522778	82,906,203	1.13 E-06	*CSN3* (Casein kappa)	GW	Litter size
8	snp12220-scaffold1455-267538	86,149,446	4.85E-07	-	GW	Beard
9	snp22971-scaffold2290-1371626	73,207,137	1.26E-06	*KATNA1* (katanin catalytic subunit A1)	CW	Wattle
15	snp36752-scaffold445-2834542	32,032,550	7.10E-06	*CLMP* (CXADR like membrane protein)	CW	Black dorsal line
17	snp46821-scaffold653-2196067	41,684,089	4.98E-06	*GLRB* (glycine receptor beta); *GRIA2* (glutamate ionotropic receptor AMPA type subunit 2)	CW	Hind leg hair
18	snp41877-scaffold546-944746	9,951,450	1.45E-06	*KCNG4* (potassium voltage-gated channel modifier subfamily G member 4)	CW	Beard length
18	snp56013-scaffold873-22716	14,190,132	1.80E-08	*TCF25* (transcription factor 25)	GW	Black dorsal line
20	snp49800-scaffold711-1045536	20,243,209	1.24E-06	*PDE4D* (phosphodiesterase 4D)	CW	Coat color
24	snp7674-scaffold1277-840997	48,881,713	5.64 E-07	*DYM* (Golgi-associated protein Dymeclin)	GW	Coat color
24	snp34951-scaffold417-602356	54,582,061	9.37E-06	Nearby *TCF4* (transcription factor 4)	CW	Litter size
26	snp11508-scaffold142-1990450	23,574,535	2.18 E-07	*SORCS3* (sortilin-related VPS10 domain-containing receptor 3)	GW	Coat color
28	snp39874-scaffold509-5956135	26,817,504	1.66 E-05	Nearby *PLA2G12B* (phospholipase A2 group XIIB), *OIT3* (oncoprotein induced transcript 3)	CW	Beard
28	snp13199-scaffold1506-389590	1,531,694	1.43E-05	*PGBD5* (piggyBac transposable element-derived protein 5)	CW	Hind leg hair

Chr, Chromosome; SNP, single nucleotide polymorphism; GW, genome-wide significance; CW, chromosome-wide significance.

## SUPPLEMENTARY MATERIAL

**Supplementary Figure S1.** Q-Q plots for nine reproduction and morphological traits in three Chinese native goat breeds.
