# Supplementary figures and images for: Genome-wide association analysis of nine reproduction and morphological traits in three goat breeds from Southern China
Source: Anim Biosci. 2022 Jun 24;36(2):191–9. doi: 10.5713/ab.21.0577 (PMC9834730; doi:10.5713/ab.21.0577)

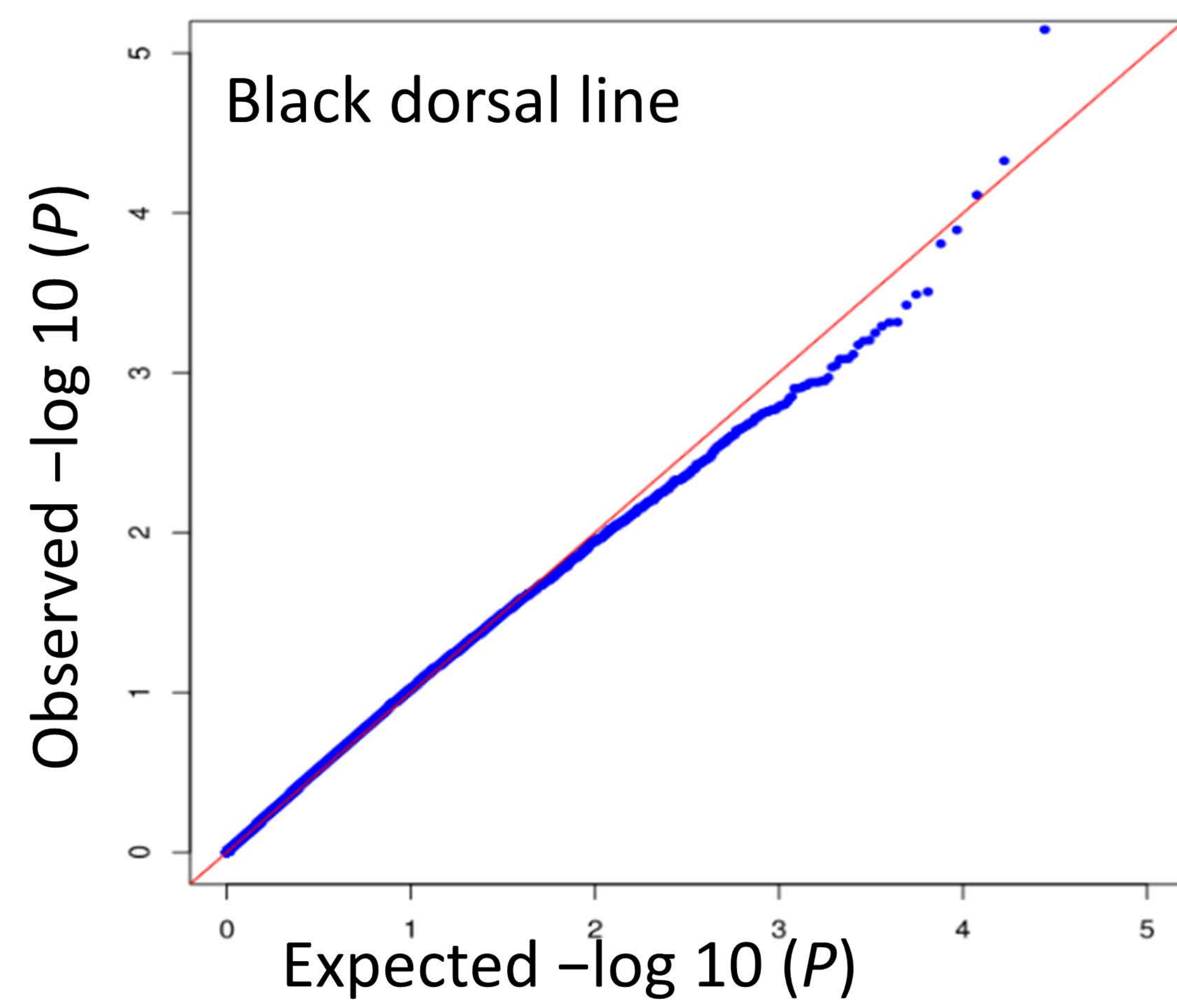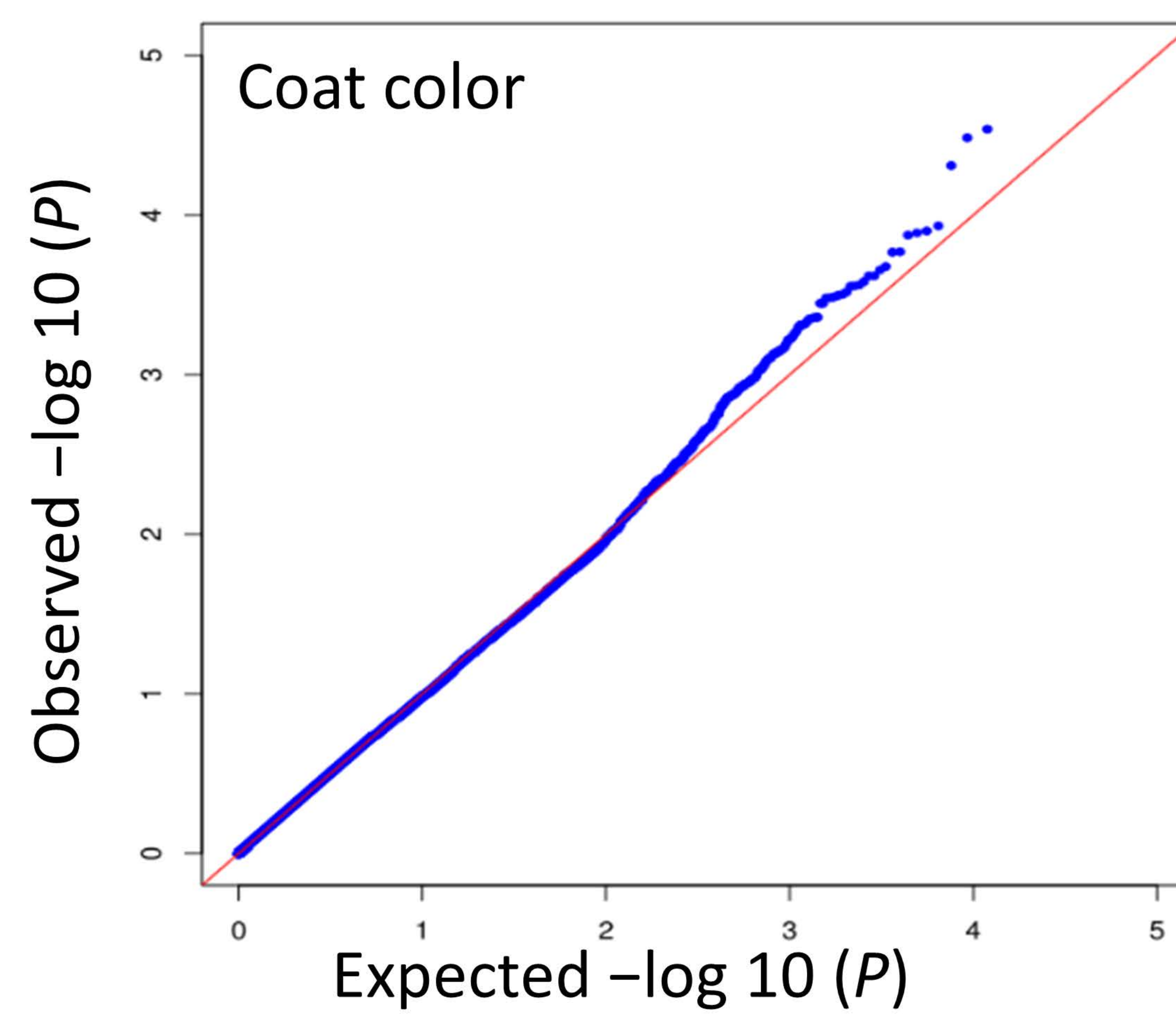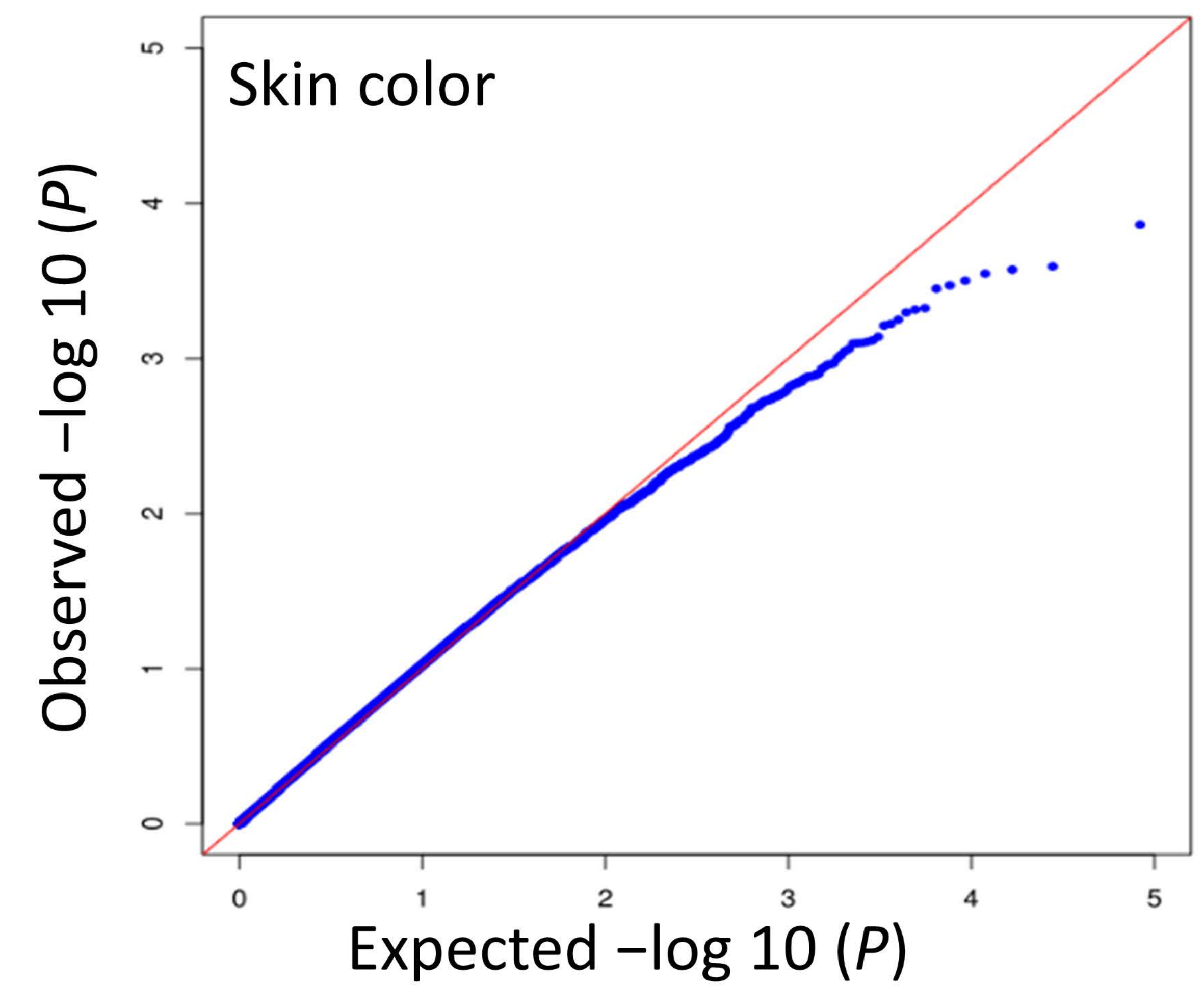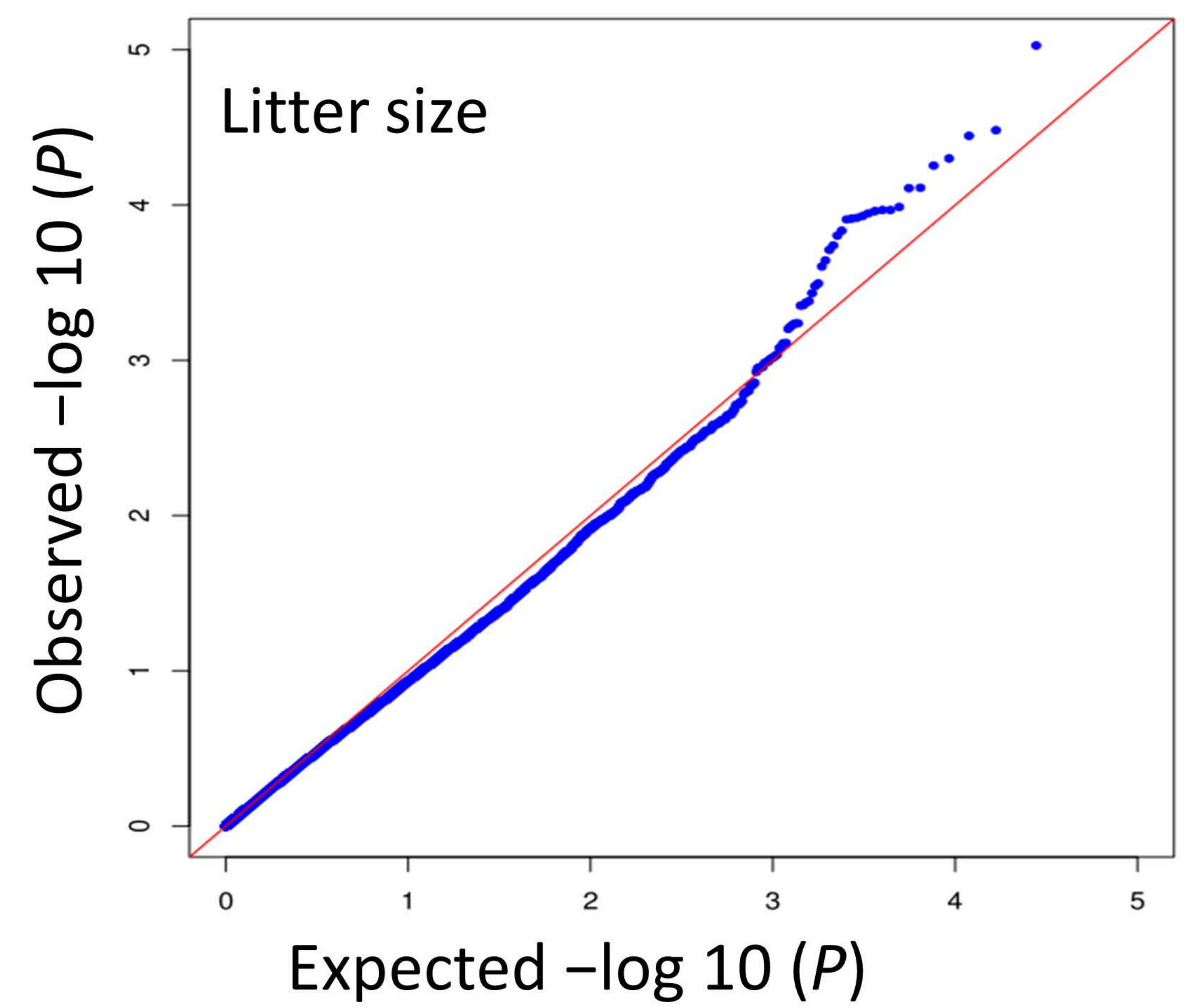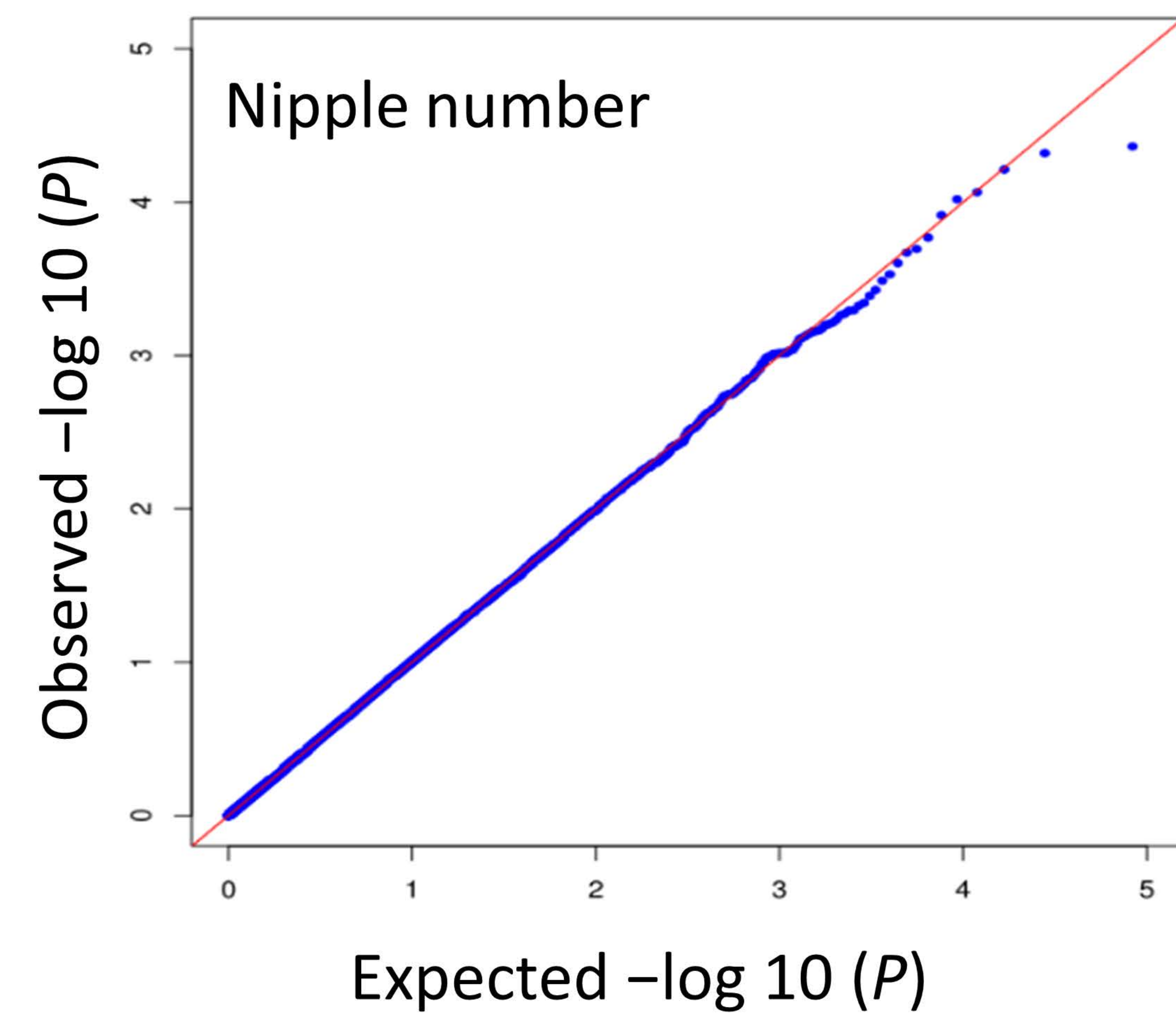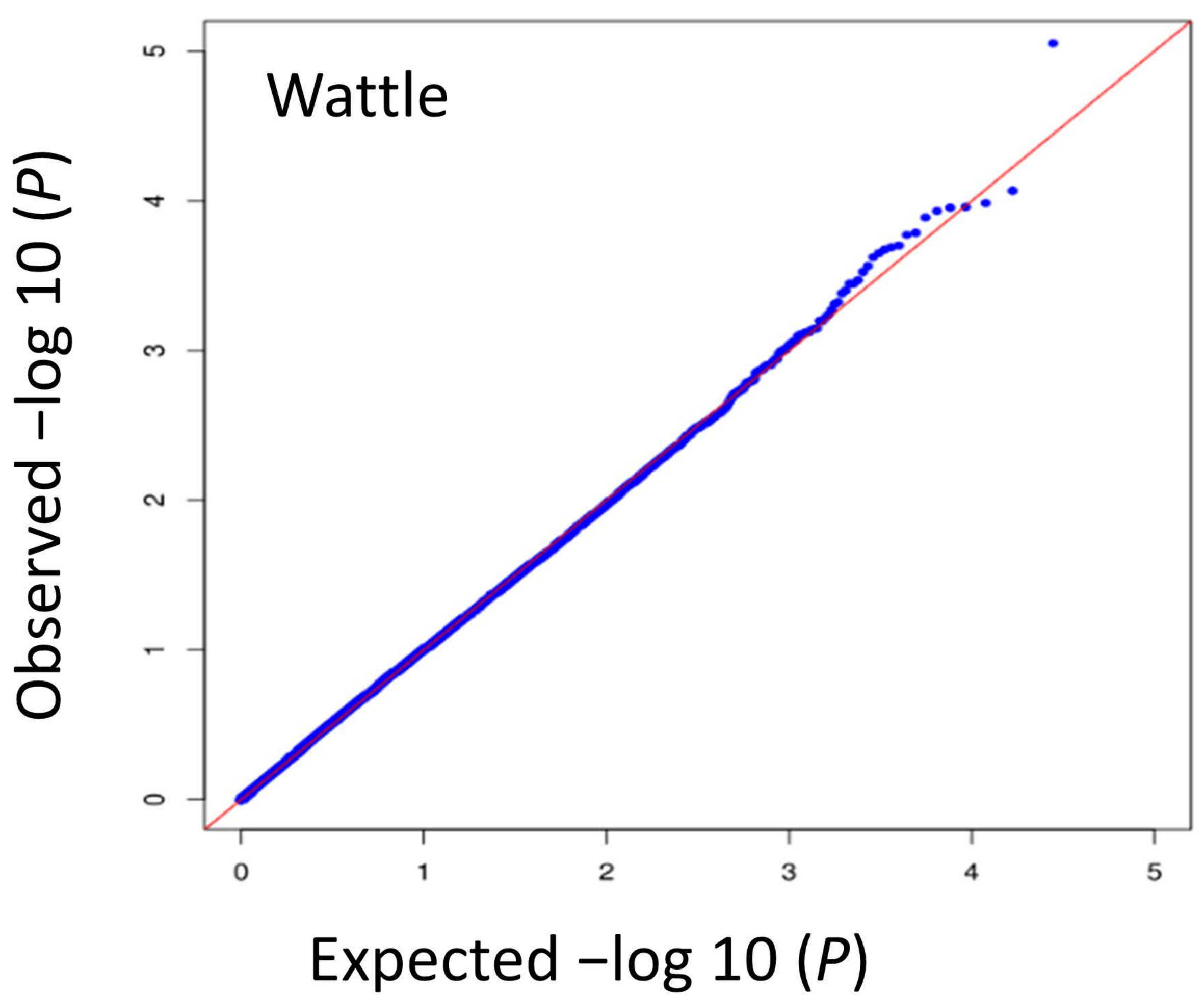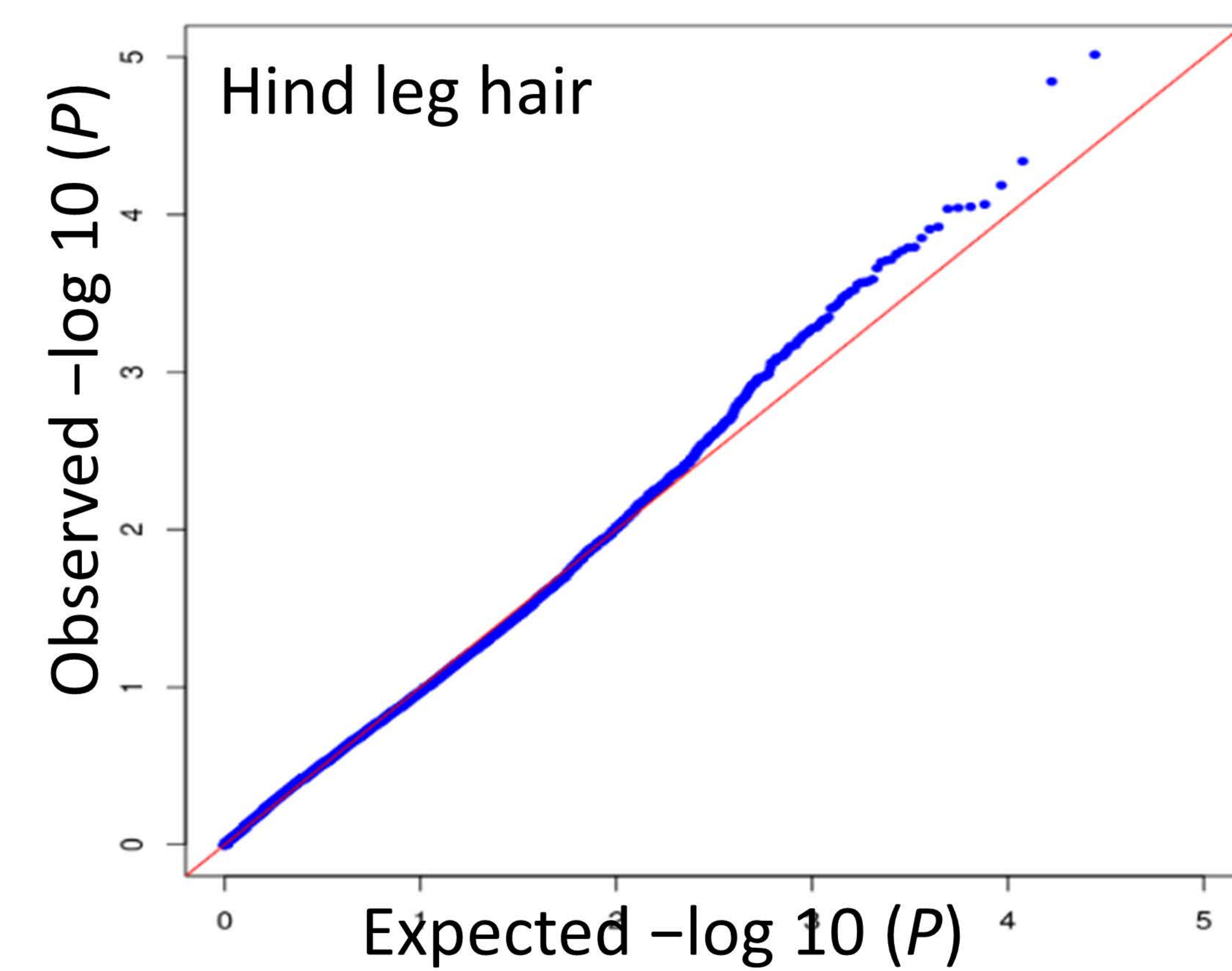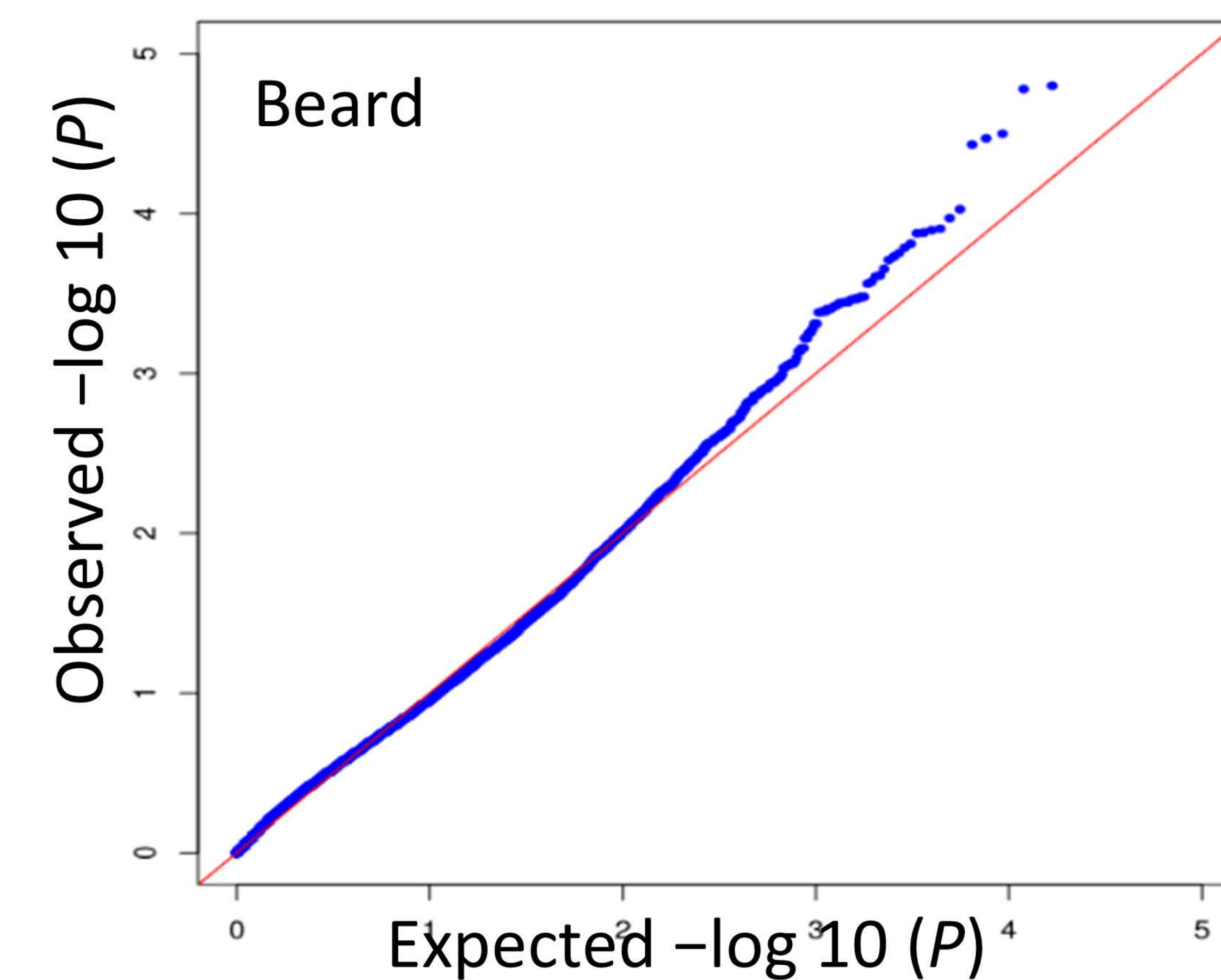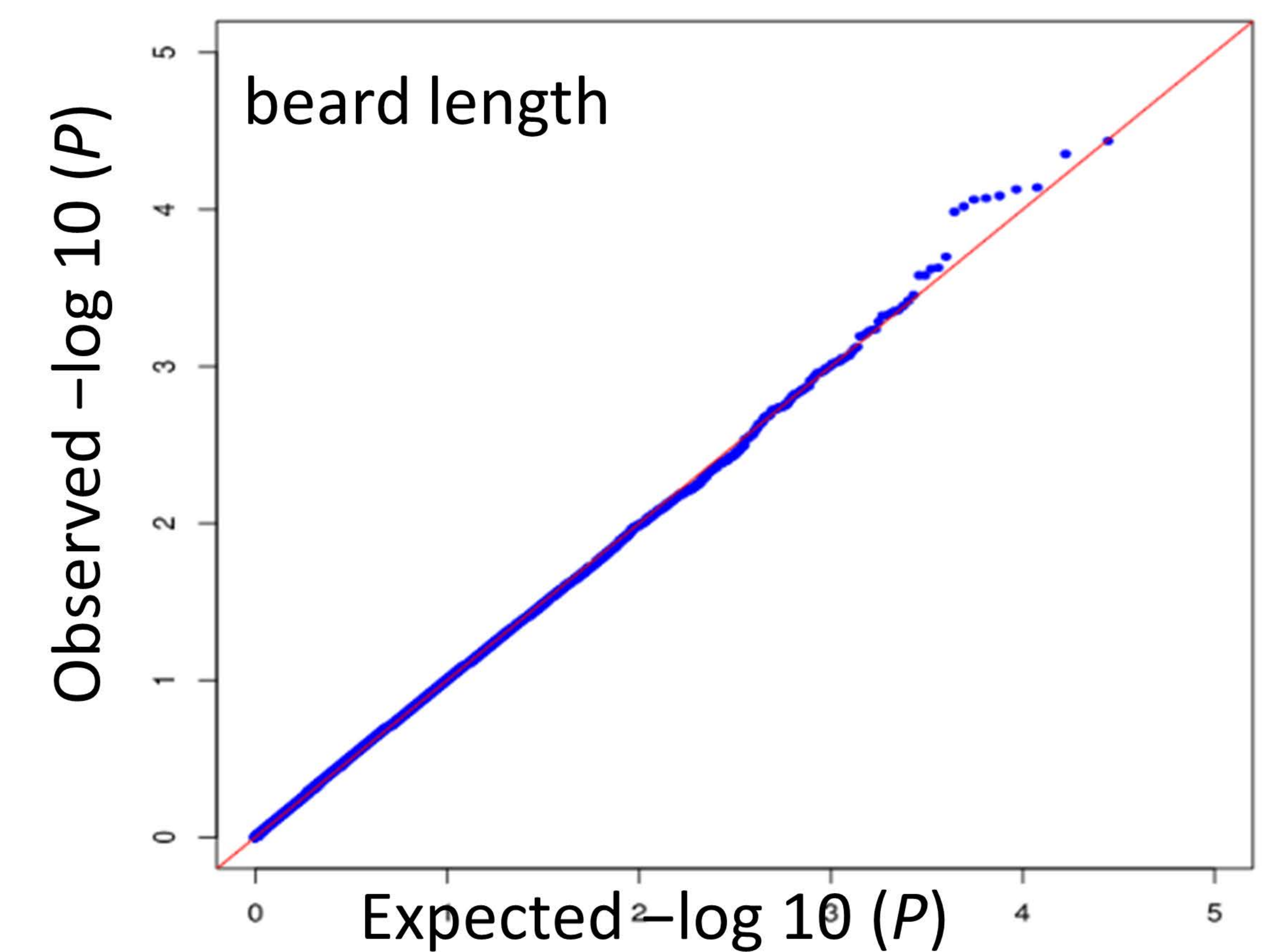

Supplement: Supplementary file 1 [file ab-21-0577-suppl.pdf]
